# Similarities of *Drosophila rab* GTPases Based on Expression Profiling: Completion and Analysis of the *rab*-Gal4 Kit

**DOI:** 10.1371/journal.pone.0040912

**Published:** 2012-07-23

**Authors:** Eugene Jennifer Jin, Chih-Chiang Chan, Egemen Agi, Smita Cherry, Elizabeth Hanacik, Michael Buszczak, P. Robin Hiesinger

**Affiliations:** 1 Department of Physiology, Medical Center, University of Texas Southwestern, Dallas, Texas, United States of America; 2 Department of Molecular Biology, Medical Center, University of Texas Southwestern, Dallas, Texas, United States of America; 3 Green Center for Systems Biology, Medical Center, University of Texas Southwestern, Dallas, Texas, United States of America; 4 UT Southwestern Green Fellow Program, University of Texas at Dallas, Dallas, Texas, United States of America; VIB and KU Leuven, Belgium

## Abstract

We recently generated *rab*-Gal4 lines for 25 of 29 predicted *Drosophila rab* GTPases. These lines provide tools for the expression of reporters, mutant *rab* variants or other genes, under control of the regulatory elements of individual *rab* loci. Here, we report the generation and characterization of the remaining four *rab*-Gal4 lines. Based on the completed ‘*rab*-Gal4 kit’ we performed a comparative analysis of the cellular and subcellular expression of all *rab* GTPases. This analysis includes the cellular expression patterns in characterized neuronal and non-neuronal cells and tissues, the subcellular localization of wild type, constitutively active and dominant negative *rab* GTPases and colocalization with known intracellular compartment markers. Our comparative analysis identifies all Rab GTPases that are expressed in the same cells and localize to the same intracellular compartments. Remarkably, similarities based on these criteria are typically not predicted by primary sequence homology. Hence, our findings provide an alternative basis to assess potential roles and redundancies based on expression in developing and adult cell types, compartment identity and subcellular localization.

## Introduction

Rab GTPases regulate intracellular membrane trafficking in all eukaryotic cells [1], [2], [3]. Several Rab GTPases have become standard markers for specific subcellular membrane compartments, yet the function of the majority of *rab* GTPases is still unknown [2], [4], [5]. Mutations in *rab* genes and their regulators cause several hereditary and neurological diseases including Griscelli syndrome (Rab27), Charcot-Marie-Tooth type 2B disease (Rab7), Warburg Micro Syndrome (a GTPase activating protein for Rab3), X-linked mental retardation (RabGDI - a Rab GTP dissociation inhibitor) and Hermansky-Pudlak syndrome (a Rab geranylgeranyl transferase) [6], [7], [8], [9]. Rab8-dependent trafficking underlies Bardet-Biedl syndrome, which causes retinopathy and blindness [10]. In *Drosophila*, post-Golgi trafficking of rhodopsin [11] and guidance receptors during brain wiring [12] depends on Rab11. Lastly, active zone assembly at synapses requires Rab3, the best known neuronal Rab GTPase [13].

The human genome contains at least 60 and maybe more than 70 *rab* genes [14], [15], [16]. The *Drosophila* genome contains 33 potential *rab* GTPase loci based on primary sequence homology, 23 of which have direct orthologs in humans with at least 50% protein similarity [15], [16], [17], [18]. Four of the 33 loci are 99% identical to recent evolutionary duplications in a cluster of six potential *rab* loci in a small interval on the X chromosome at cytological location 9C–F [19], leading us to predict a total of 29 potential *rab* genes in *Drosophila* [17]. We have recently performed a systematic profiling effort for 25 of these loci [17]. The two other conserved loci in this X chromosomal cluster (RabX2 and RabX3) were the only predicted *rab* genes for which we found no expression [17]. Hence, the total number of functional *rab* loci in *Drosophila* may only be 27. We have previously characterized 23 of these 27 through the analysis of *rab*-Gal4 driver lines [17]. The Gal4/UAS system is the most widely used binary expression system in *Drosophila* [20], [21]. We used recombineering to precisely insert the Gal4 open reading frame into the start codon site of each *rab* GTPase within a large (20–50 kb) genomic fragment [17], [22]. The large genomic fragments are predicted to preserve all regulatory elements, thus yielding Gal4 lines that can be used to drive fluorescent reporters or fluorescently tagged variants of the Rabs themselves as wild type, constitutively active or dominant negative proteins. Several of the original 23 *rab*-Gal4 lines were verified using antibodies, proteins traps or rescue experiments [17].

Here, we report cellular and subcellular expression patterns of the four remaining *rab*-Gal4 lines, namely *rab30*, *rab40*, *rabX5* and *rabX6*. All four are novel *rab* GTPases of largely unknown function. In agreement with our recent findings that up to half of all *rabs* are either neuron-specific or highly enriched in neurons, we found that *rabX5*-Gal4 and *rabX6*-Gal4 are novel neuron/glia-specific Gal4 lines, whereas *rab30*-Gal4 expresses ubiquitously and *rab40* only very weakly. In addition to obtaining cellular expression data, we analyzed subcellular localization by expressing YFP-tagged wild type, constitutively active (GTP-bound) and dominant negative (GDP-bound) YFP-tagged Rab proteins under their own regulatory elements. Finally, we performed a preliminary characterization of the subcellular compartments marked by these four novel Rab proteins.

The completion of the ‘*rab*-Gal4 kit’ makes it possible to perform a comprehensive comparison of cellular and subcellular localization features of all *Drosophila* Rabs. Homology is an important indicator for potential redundancies, especially in a gene family with a common ancestor. However, in order to have the potential of a redundant function *in vivo*, the proteins should be expressed in the same cell at the same time. In the case of Rab GTPases, localization to the same intracellular compartment is a further likely prerequisite for redundancy. With this idea in mind, we present here a comparative analysis of the 27 predicted fly *rab* GTPases for 33 criteria that include expression in specific tissues or cells and subcellular localization of the wild type, dominant negative and constitutively active proteins. Our findings indicate that protein sequence similarity in many cases poorly predicts which Rabs share common expression and localization patterns. These analyses will serve as a guide to assess which *rabs* carry out specific functions based on their cellular and subcellular localization.

## Materials and Methods

### Molecular Biology, Recombineering, and *Drosophila* Genetics

**Figure 1:**
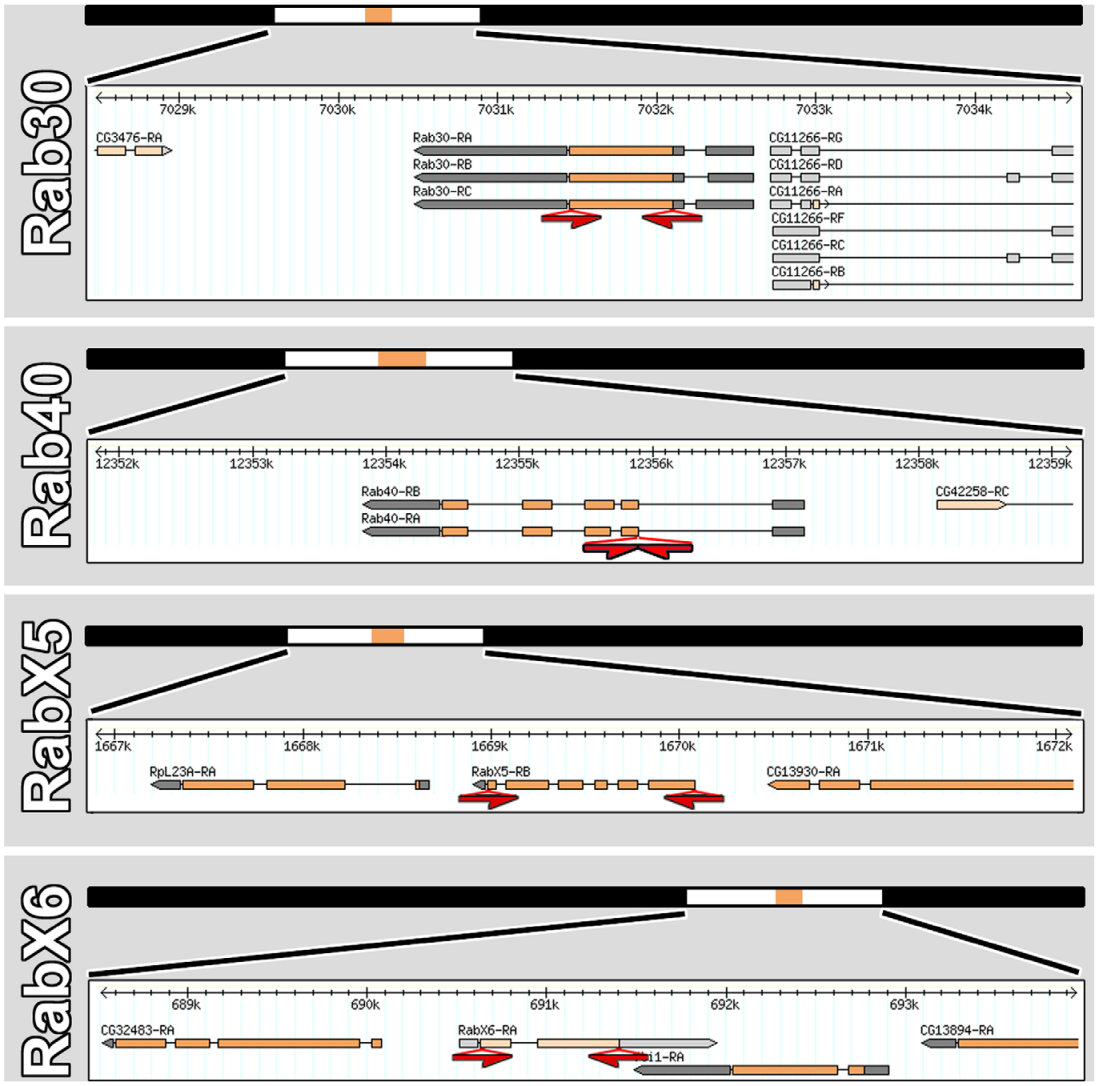
Targeting vector design for *rab30*-Gal4, *rab40*-Gal4, *rabX5*-Gal4 and *rabX6*-Gal4. 20–22 kb genomic regions (black bars) were recombineered from bacterial artificial chromosomes (BACs) into attB-P[acman]-KO [17], [22]. Regions of a few kb are shown at higher resolution to reveal the structures of rab loci within these genomic regions. Sequences between red arrows were replaces with a Gal4 knock-in cassette [17]. For expression analyses, transgenic flies with the targeting vectors inserted in the same landing site were used.

We previously generated 50–55 kb targeting vectors for *rab30, rab40, rabX5* and *rabX6*, but failed to obtain transformants after injection of more than 1,500 embryos each [17]. For the generation of new targeting vectors we chose smaller genomic regions which include sequences 15 kb upstream and 5 kb downstream of the *rab* loci (Fig. 1). In addition, we applied small improvements to the recombineering protocol and verification of the final targeting cassette. These modifications include PCR and sequencing verifications for the precision of the Gal4 knock-ins as described recently [22]. Finally, transformation efficiency is greatly enhanced if the DNA of the large vectors is ‘maxi’-prepped at the place of injection, i.e. without excessive handling or shipping, and injected with minimal delay time.

Complete open reading frames (ORFs) were replaced as before for *rab30, rabX5* and *rabX6*. In contrast to these three *rab* loci, *rab40* contains long introns. We therefore replaced only the short coding regions starting with the ATG to the end of the ATG-containing exon (Fig. 1). All vectors were verified by sequencing. Transgenic fly strains were established using standard procedures at Rainbow Transgenics, Inc. All vectors were inserted in the same landing site attP-3B (Bloomington Stock #24871) to generate the *rab*-Gal4 transgenic flies. The new rab-Gal4 lines were crossed to UAS-CD8-GFP as well as the respective UAS-YFP-Rabs (wild type, constitutively active and dominant negative) precisely as in the original study [17], [18]. All flies were kept at 25 C.

### Immunohistochemistry, Microscopy, and Image Processing

Larval brains and tissues, pupal brains and adult brains were dissected and prepared for confocal microscopy as previously reported [23]. The tissues were fixed in phosphate buffered saline (PBS) with 3.5% formaldehyde for 15 min and washed in PBS with 0.4% Triton X-100. High-resolution light microscopy was performed using a Confocal Microscope (Leica SP5). Imaging data was processed and quantified using Amira 5.2 (Indeed, Berlin, Germany) and Adobe Photoshop CS4 as described in [24]. The following antibodies were used at 1∶500: rabbit anti-rab5, rabbit anti-rab7 [25], mouse anti-rab11. A mouse monoclonal antibody against CSP was used at 1∶50.

### Pair-wise Similarity Analyses

The presence or absence of expression or colocalization was determined manually in high-resolution 3D confocal datasets. For the pair-wise comparisons the data was binarized, i.e. any level of expression or colocalization was counted as 1, each absence as 0. Each pair of the 27 rabs was separately compared for expression in 25 cell types or brain structures (Table 1) as well as for eight subcellular localization criteria (Table 2).

**Table 1:** Similarities of *rab*-Gal4 expression patterns based on expression patterns.

SIM_Rab_	Rab1	Rab2	Rab3	Rab4	Rab5	Rab6	Rab7	Rab8	Rab9	Rab10	Rab11	Rab14	Rab18	Rab19	Rab21	Rab23	Rab26	Rab27	Rab30	Rab32	Rab35	Rab39	Rab40	RabX1	RabX4	RabX5	RabX6
** = <100%**	2, 5, 6, 8, 11, **35**, 39	1, 5, 6, 8, 11, 35, 39		18	1, 2, 6, 8, 11, 35, 39	1, 2, **5**, 8, 11, 35, 39		1, 2, 5, 6, 11, 35, 39			1, **2**, 5, 6, 8, 35, 39		4								**1**, 2, 5, 6, 8, 11, 39	1, 2, 5, 6, 8, 11, 35					
** = <96%**	4, 7, 14, 18	4, 7, **14**, 18	X4	1, **2**, 5, 6, 8, 10, 11, 35, 39	4, 7, 14, 18	4, 7, 14, 18	1, 2, 5, 6, 8, 11, 35, 39	4, 7, 14, 18		4, 18	**4**, 7, **14**, 18	1, **2**, 5, 6, 8, 11, 35, 39	1, 2, 5, 6, 8, 10, 11, 35, 39								4, 7, 14, 18	4, 7, 14, 18			3		
** = <92%**	10	10		7, **14**	10	10	4, 14, 18	**10**		1, 2, 5, 6, **8**, 11, 35, 39	10	4, 7, 18	7, 14								10	10					
** = <88%**							10			7, 14		10															
** = <84%**				X1			X1						X1											4, 7, 18			
** = <80%**	X1	X1			X1	X1		X1		X1	X1										X1	X1		1, 2, 5, 6, 8, 10, 11, 35, 39			
** = <76%**							X4			30		X1		X1, X6					10					14, 19	7		19
** = <72%**	X4	X4	7, 14	21, 30	X4	X4	3, 21	X4		X6	X4	3, 30	21, 30		4, 7, 18				4, 14, 18, X1		X4	X4		30, X6	1, 2, 5, 6, **8**, 11, 35, 39		10, X1
** = <68%**	3, 21, 30	3, 21, 30	1, 2, 5, 6, 8, 11, 35, 39	23, X4, X6	3, **21**, 30	3, **21**, 30	X6	3, 21, 30		21	3, 21, 30	X4	23, X4, X6	X4	1, 2, **5**, 6, 8, 10, 11, 35, 39, X6	4, 18, 30			1, 2, 5, 6, 8, 11, 23, 35, 39, X6		3, 21, 30	3, 21, 30		X4	4, 14, 18, 19, X1		4, 7, 18, 21, 30
** = <64%**	23, X6	23, X6	4, 18, X1	3, 19	23, X6	23, X6	19, 30	23, X6		23, X4	23, X6	21	3, 19	4, 7, 18	14, X1	1, 2, 5, 6, 8, 10, 11, 35, 39, X1			7		23, X6	23, **X6**		3, 21, 23	**10**		1, 2, 5, 6, 8, 11, 35, **39**
** = <60%**	19	19	10, 19, 26		19	19	**9**, 23	19	**7**, 14	3, 19	19	9, 23, X6		1, 2, 3, 5, 6, 8, 10, 11, 26, **30**, 35, 39		7, 14	**3**, 19		**19**		19	19					14
** = <56%**	9	9			9	9		9	1, 2, 5, 6, 8, 10, 11, 19, 30, 35, 39, X1	9	9	19		9, 14, 21, 23	19	19, X6	X4		9		9	9		9	26, X6		23, X4
** = <52%**			X6	9					4, 18, X6				9		23, 30	21	32, X1		21	26				26			3, 9
** = <48%**			9, 30						3, X4						X4	26, X4	23, X6		3, X4						9, 21, 23, 30		26
** = <44%**			21, 23, **27**	26			26		21, 23			26	26		3, 9, 26	3, 9	4, 7, 14, 18, 21, **27**, 30	**3**, 26	26								
** = <40%**	26	26			26	26		26		26	26						1, 2, 5, 6, 8, 10, 11, 35, 39	X4			26	26			27		
** = <36%**														32				32, X1		19, 27				27			
** = <32%**			32	27			27					27	27	27		32		4, 7, 14, 18, 19		3, 23							
** = <28%**	27	27			27	27		27	26	27	27					27	9	1, 2, 5, 6, 8, 10, 11, 23, 35, 39		X1, X4	27	27		32	32		
** = <24%**	32, X5, 40	32, X5, 40	X5, 40	32, X5, 40	32, X5, 40	32, X5, 40	32, X5, 40	32, X5, 40	27,32, X5, 40	32, X5, 40	32, X5, 40	32, X5, 40	**32,** X5**,** 40	X5, 40	32, 27, 40, X5	**X5,** 40	40, X5	9, X6, 21, 30, X5, 40	32, 27, X5, 40	1, 2, 4, 5, 6, 7, 8, 9, 10, 11, 14, **18**, 21, 30, 35, 39, 40, X5, X6	32, X5, 40	32, X5, 40	1, 2, 3, 4, 5, 6, 7, **8**, 9, **10**, 11, 14, 18, 19, 21, 23, 26, 27, 30, 32, 35, 39, 40, X1, **X4**, X5, X6	X5, 40	X5, 40	27, 3, 4, 7, 9, 10, 14, 18, 19, **23**, 30, X1, X4, X6, 1, 2, 5, 6, 8,11, 35, 39, 21, 26, 32, 40	27, 32, X5, 40

Summary of pair-wise comparisons of *rab*-Gal4 expression patterns for all 27 *rab* GTPases. Similarity was calculated as described in the Materials and Methods. Closest protein homologs are highlighted in bold.

Similarities between two *rabs* were calculated separately for the 25 cellular and 8 subcellular criteria. Only criteria in which at least one *rab* was positive were considered. Hence, a ‘1’ for both *rabs* was counted as a similarity, a ‘1’ and a ‘0’ as a discrepancy and a ‘0’ for both was disregarded. This latter rule prevents a scenario where two *rabs* that have no expression or colocalization in common might otherwise appear similar solely based on common absence of expression of colocalization. Similarity Sim_rab_ for two *rabs*, rabA and rabB, was therefore calculated as follows:
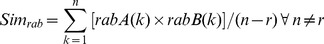
with n  =  total number of criteria (25 for cellular expression in Table 1 and eight for subcellular criteria in Table 2); rabA(k) and rabB(k)  =  binary value of presence (1) or absence (0) of criterion number k; r  =  total number of criteria where both rabA and rabB are absent. The resulting similarities are shown in Tables 1 and 2.

**Table 2:** Similarities of Rab GTPases based on subcellular localization features.

SIM_Rab_	Rab1	Rab2	Rab3	Rab4	Rab5	Rab6	Rab7	Rab8	Rab9	Rab10	Rab11	Rab14	Rab18	Rab19	Rab21	Rab23	Rab26	Rab27	Rab30	Rab32	Rab35	Rab39	Rab40	RabX1	RabX4	RabX5	RabX6
** = <100%**	6, X6	4	**27**	**2**		1, X6					X1			21, X4	19, X4			**3**	32	30				11	19, 21		1, 6
** = <87.5%**								**10**, 23		**8**	23					8, 11, 30, 32, 35, X1			23	23	23			23			
** = <75%**	2, 4, 14, 19, 21, X4	1, 5, 6, 7, 11, 35, X1, X6	30, 32, 35	1, 5, 6, 7, 11, 35, X1, X6	2, 4	2, 4, 14, 19, **21**, X4	2, 4, 14	9, 19, 21, X4	8, 30, 32	23	**2**, **4**, 19, 21, 26, X4	1, 6, 7, 39, X6		1, 6, 8, 11, 39, X1, X6	1, 6, 8, 11, 39, X1, X6	10	11, 30, 32, X1	30, 32, 35	3, 9, 26, 27	3, 9, 26, 27	2, 3, 4, 27	14, 19, 21, X4		2, 4, 19, 21, 26, X4	1, 6, **8**, 11, 39, X1, X6		2, 4, 14, 19, 21, X4
** = <62.5%**		23	23	23	7, 11, 35, X1		5, 11, 35, 39, X1	11, 30, 32, 35, 39, X1	10, 23	9, 19, 21, X4	5, 7, 8, 30, 32, 35, 39			10, 23	10, 23	2, 3, 4, 9, 19, 21, 26, 27, X4	23	23	8, 11, 35, X1	8, 11, 35, X1	5, 7, 8, 11, 30, 32, X1	7, 8, 11, X1		5, 7, 8, 30, 32, 35, 39	**10**, 23		
** = <50%**	5, 7, 8, 10, 11, 23, **35**, 39, 40, X1	3, 8, **14**, 19, 21, 26, 27, 30, 32, 39, X4	2, 4, 5, 7, 8, 9, 11, 26, X1	3, 8, **14**, 19, 21, 26, 27, 30, 32, 39, X4	1, 3, 6, 14, 18, 19, **21**, 23, 26, 27, X4, X6	**5**, 7, 8, 10, 11, 23, 35, 39, 40, X1	1, 3, 6, 19, 21, 23, 26, 27, X4, X6	1, 2, 3, 4, 6, 7, 14, 26, 27, X6	3, 11, 19, 21, 26, 27, 35, 39, X1, X4	1, 6, 11, 30, 32, 35, 39, X1, X5, X6	1, 3, 6, 9, 10, **14**, 27, X6	**2**, 4, 5, 8, 11, 19, 21, 35, X1, X4	5	2, 4, 5, 7, 9, 14, 26, **30**, 32, 35	2, 4, **5**, 7, 9, 14, 26, 30, 32, 35	1, 5, 6, 7, 39, X6	2, **3**, 4, 5, 7, 8, 9, 19, 21, **27**, 35, 39, X4	2, 4, 5, 7, 8, 9, 11, 26, X1	2, 4, 10, **19**, 21, X4	2, 4, 10, 19, 21, X4	**1**, 6, 9, 10, 14, 19, 21, 26, X4, X6	1, 2, 4, 6, 9, 10, 23, 26, **X6**	1, 6, X5, X6	1, 3, 6, 9, 10, 14, 27, X6	2, 4, 5, 7, 9, 14, 26, 30, 32, 35	10, 40	5, 7, 8, 10, 11, 23, 35, **39**, 40, X1
** = <37.5%**	18, X5	10, 18, 40	10, 40	10, 40	8, 10, 30, 32, 39	18, X5	8, 10, 30, 32	5, 7	**40**	2, 3, 4, 5, 7, 14, 26, 27		10, 23, 40	1, 2, 6, X6	40	40	14	10, 40	10, 40	5, 7, 39	5, 7, 39	39	5, 30, 32, 35	2, 3, 4, 9, 14, 19, 21, 26, 27, **X4**		40	1, 6, X6	18, X5
** = <25%**	3, 9, 26, 27, 30, 32	9, X5	1, 6, 14, 19, 21, 39, X4, X5, X6	9, 18, X5	9, 40, X5	3, 9, 26, 27, 30, 32	**9**, 18, 40, X5	18, 40, X5	1, 2, 4, 5, 6, **7**, 14, X5, X6	18, 40	18, 40, X5	3, 9, 18, 26, 27, 30, 32, X5	4, 7, 8, 10, 11, 14, 19, 21, 23, 35, 39, X1, X4	3, 18, 27, X5	3, 18, 27, X5	18, 40, **X5**	1, 6, 14, X5, X6	1, 6, 14, 19, 21, 39, X4, X5, X6	1, 6, 14, 40, X5, X6	1, 6, 14, 40, X5, X6	18, 40, X5	3, 18, 27, 40, X5	5, 7, **8**, **10**, 11, 23, 30, 32, 35, 39, X1	18, 40, X5	3, 18, 27, X5	2, 3, 4, 5, 7, 8, 9, 11, 14, 19, 21, **23**, 26, 27, 30, 32, 35, 39, X1, X4	3, 9, 26, 27, 30, 32
** = <12.5%**			18						18				3, 9, 26, 27, 30, **32**, 40, X5				18	18	18	**18**			18			18	

Pair-wise analyses for all 27 *rabs* were performed, and their similarity was determined as described in Materials and Methods. Bold *rabs* indicate the closest homologous Rabs in protein sequence alignments based on Figure 1 in [18] and Figure S3 in [17]. In cases of two or three pone.0040912.g006.tifclose homologs.

## Results

### Completion of the ‘*rab*-Gal4 kit’

We recently presented a first systematic effort towards a functional characterization of all *rab* GTPases in *Drosophila* [17]. We developed a streamlined cloning strategy for the generation of *rab*-Gal4 lines as versatile tools that can be used to express any gene under control of the endogenous regulatory elements of a particular *rab* locus [17], [22], [26]. In particular, the availability of a complementary kit of UAS-YFP-Rab lines in combination with the *rab*-Gal4 lines offers the opportunity to express wild type (WT), constitutively active (CA, GTP-bound) and dominant negative (DN, GDP-bound) Rabs under their own regulatory elements in wild type or mutant backgrounds [17], [18]. The cloning strategy underlying the generation of the *rab*-Gal4 lines is based on P[acman] technology, an implementation of bacterial artificial chromosome (BAC) recombineering in *Drosophila* [27], [28]. We inserted Gal4 cassettes into large genomic fragments (20–55 kb) that are predicted to contain all regulatory elements of individual *rab* loci in order to ensure faithful replication of the endogenous expression patterns. However, the transformation of these large vectors proved difficult in individual cases. Out of 29 *rab* loci, we originally failed to obtain transformants for four: *rab30, rab40, rabX5* and *rabX6*. These problems were likely related to these particular genomic sequences; however, we cannot exclude other issues with the original 50–55 kb transformation vectors, since we did not sequence them in their entirety. Since the publication of the first ‘*rab*-Gal4 kit’, we have improved all steps of the technology including the verification of the correct recombineering products, the transformation and the possibility to mobilize the targeting cassette from the original landing site to generate a knock-in in the endogenous locus [22]. Some modifications are very simple, but drastically improve specific steps, e.g. the avoidance of excessive handling and time delays between DNA preparation and injection for transformation.

The objective of the present study was to complete the ‘*rab*-Gal4 kit’ and thereby be in a position to perform a comprehensive comparison of all Rabs with respect to expression pattern, subcellular localization, intracellular compartment identity and localization behavior as WT, CA and DN proteins. We generated new Gal4 vectors for *rab30, rab40, rabX5* and *rabX6* using smaller genomic fragments as shown in Fig. 1. Specifically, we reduced the 5′ genomic region to 15 kb and the 3′ genomic sequence to 5 kb and generated transgenic flies as described [17], [22].

### Cellular Expression Profiling of the New *rab*-Gal4 Lines

**Figure 2:**
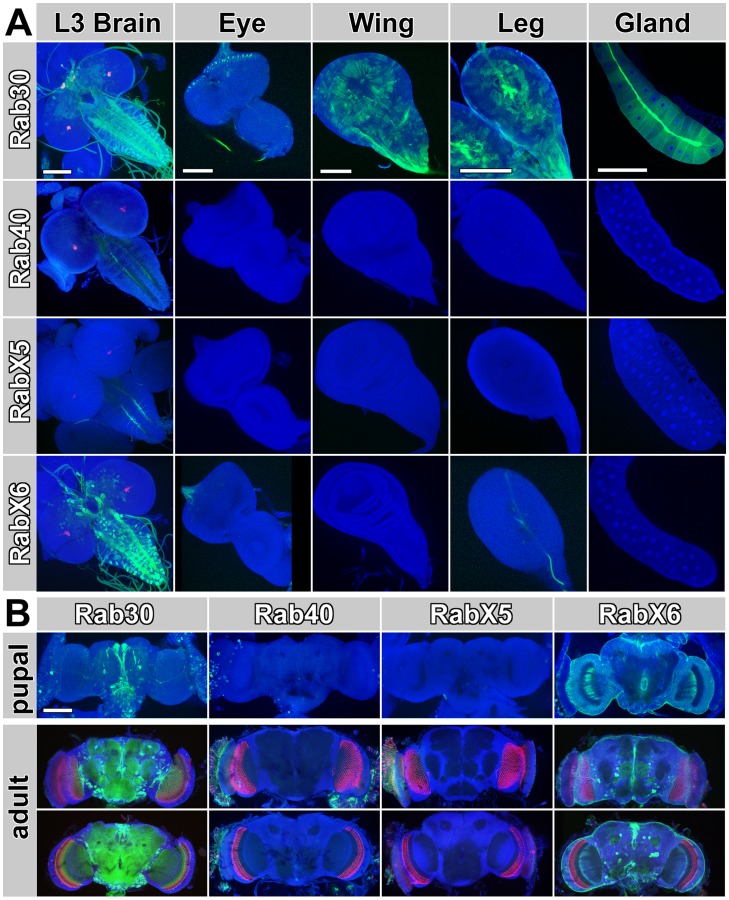
Cellular Expression Analysis of *rab30*-Gal4, *rab40*-Gal4, *rabX5*-gal4 and *rabX6*-Gal4. All rab-Gal4 lines were crossed to UAS-CD8-GFP. (A) Larval tissues showing GFP expression in green, 3×P3-RFP (positive marker of the Gal4 knock-in cassette) and nuclear Toto3 in blue. (B) Pupal (P+30% +/−5%) and 1-day adult brains (top panel: anterior; bottom panel: posterior. All scale bars represent 100 µm.

To determine the cellular expression pattern of these *rab-*Gal4 lines, we crossed them to UAS-CD8-GFP and obtained high-resolution 3D confocal datasets for the L3 larval brain, eye disc, wing disc, leg disc and salivary gland as well as P+30% pupal brains and adult brains (Fig. 2). *rab30*-Gal4 expresses ubiquitously in all or at least in most cell types. However, as observed for several other *rab*-Gal4 lines, expression levels of rab30-Gal4 vary strongly in different cell types, more so than other evenly expressing ubiquitous lines such as *rab5*-Gal4 and *rab11*-Gal4 [17]. In contrast to *rab30*, *rab40*-Gal4 expresses at very low levels and mostly below the detection limit in the imaginal discs, salivary glands as well as pupal and adult brains (Fig. 2A, B). *rabX5*-Gal4 also shows weak but specific expression in some neurons of the ventral ganglion in the larval brain (Fig. 2A). Finally, *rabX6*-Gal4 exhibits strong expression in the larval brain and neurons innervating the leg disc. Expression in non-neuronal tissues like the wing disc or salivary gland was not observed. In the eye disc and pupal brain, glial expression is most pronounced. Expression of *rabX6*-Gal4 in the adult brain is weaker and more-sparse compared to *rab30*-Gal4 and again strongest in glial cells (Fig. 2B). In summary, the new *rab*-Gal4 lines corroborate our previous observation of highly variable *rab* expression levels, especially in the nervous system [17].

### Subcellular Localization of Rab30, Rab40, RabX5 and RabX6 in Neurons

**Figure 3:**
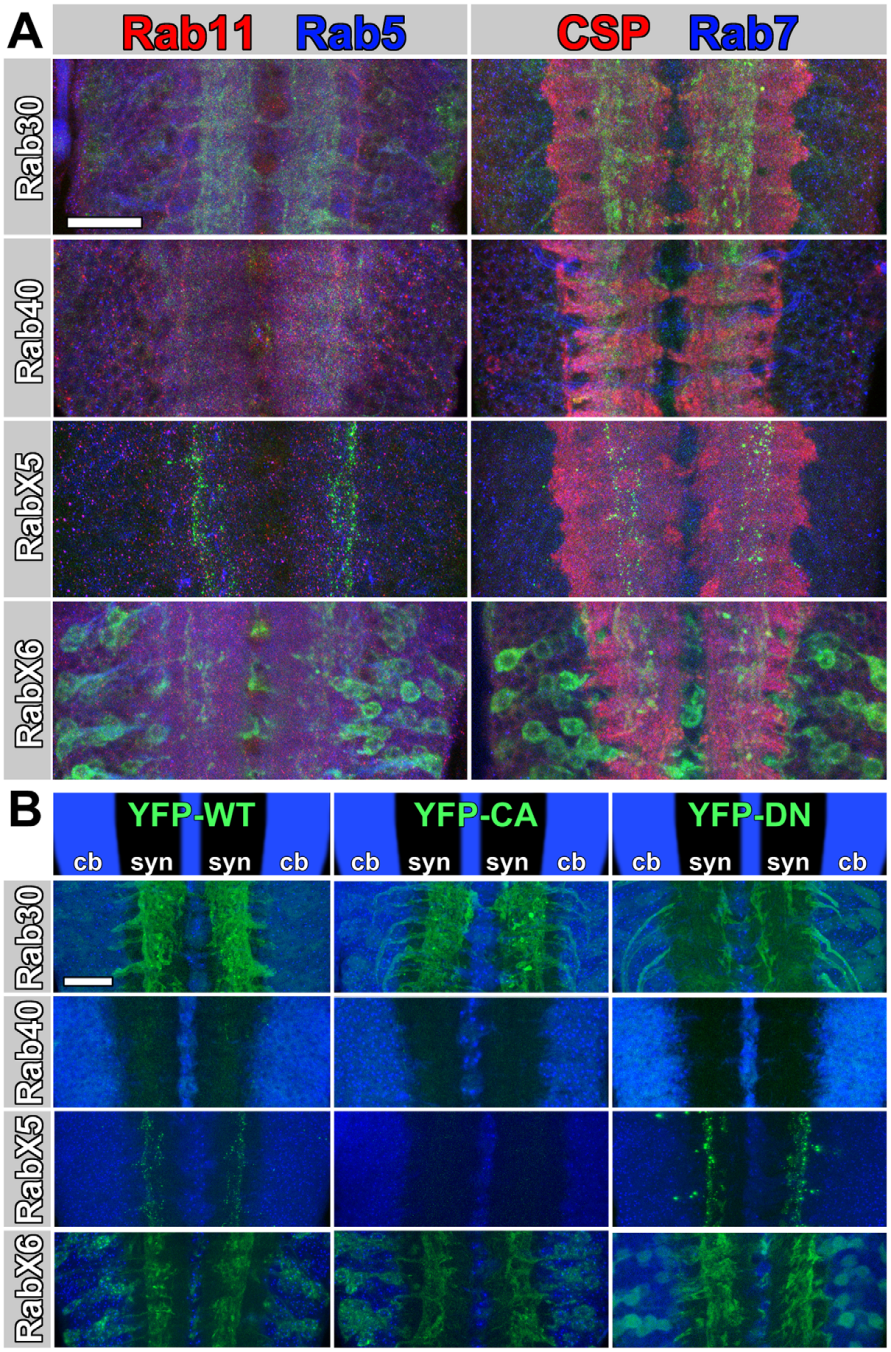
Subcellular Localization Features of YFP-Rab30, YFP-Rab40, YFP-RabX5, and YFP-RabX6. (A) Double immunolabelings of the posterior larval brain ventral ganglion at high resolution are shown for the four YFP-Rabs driven by their respective rab-Gal4 lines. Left column: YFP-Rab (green), anti-Rab11 (red, recycling endosomes), anti-Rab5 (blue, early endosomes); right column: YFP-Rab (green), anti-CSP (red, synaptic vesicles), anti-Rab7 (blue, late endosomes). Cell bodies are peripherally and synaptic neuropils centrally located. Scale bar for all panels represents 20 µm. (B) Corresponding Gal4-lines drive the expression of wild type YFP-tagged Rabs in the left column, constitutively active (GTP-bound) YFP-tagged Rabs in the middle column and dominant negative (GDP-bound) YFP-tagged Rabs in the right column. Toto-3 labels nuclei (blue). Scale bar for all panels represents 20 µm.

Next, we investigated the subcellular localization of YFP-Rab30, YFP-Rab40, YFP-RabX5 and YFP-RabX6 expressed by their respective *rab*-Gal4 lines in neurons of the larval ventral ganglion. With respect to cell body or synaptic localization Rab30 is localized in both, but stronger at synapses; Rab40 is at low levels present in both; RabX5 is specific to the synaptic region of the ventral ganglion; RabX6 is mostly in the cell bodies and to a lesser extent at synapses (Fig. 3A). Taken together with all other Rabs, RabX6 is the only neuronal/glial Rab that predominantly localizes to cell bodies and not to synapses.

To reveal the identities of subcellular compartments marked by YFP-Rab30, YFP-Rab40, YFP-RabX5, and YFP-RabX6, we co-labeled the larval brain preparations with antibodies that mark early endosomes (Rab5), late endosomes (Rab7), recycling endosomes (Rab11) and synaptic vesicles, (Cysteine-String Protein, CSP). In contrast to our previous analyses of 23 YFP-Rab proteins, none of these novel Rabs strongly colocalize with any of the markers. RabX5 and RabX6 in particular label clear subcellular structures that are not positively labeled by any of the four antibodies. YFP-Rab40 levels may have been too low for a decisive analysis. Only Rab30 showed weak and partial colocalization with both Rab11 and CSP (Fig. 3A).

Rab GTPases cycle between GTP-bound and GDP-bound forms. A complete set of constitutively active (CA) and dominant negative (DN) UAS-YFP-Rab lines has previously been generated [18]. We performed functional studies with these by again expressing each YFP-Rab protein under control of their own regulatory elements with the respective *rab*-Gal4 line. As shown in Fig. 3B, Rab30 exhibits the typical and most commonly previously observed behavior of a more diffuse, cell body biased localization of the DN, whereas the WT and CA variants mark distinct structures especially at synapses. RabX6 has a similar behavior, except that WT and CA variants mark more distinct compartments in the cell bodies that are lost with the DN variant. The Rab40 CA and DN variants were too weak to be scored with confidence. RabX5 exhibited an unusual behavior, where the DN variant exhibits increased synaptic compartments (Fig. 3B). In summary, none of the four new Rabs exhibit cellular or subcellular localization profiles that are identical to any of the previously characterized 23 Rabs.

### Similarities of *rab*-Gal4 expression patterns based on expression patterns

**Figure 4:**
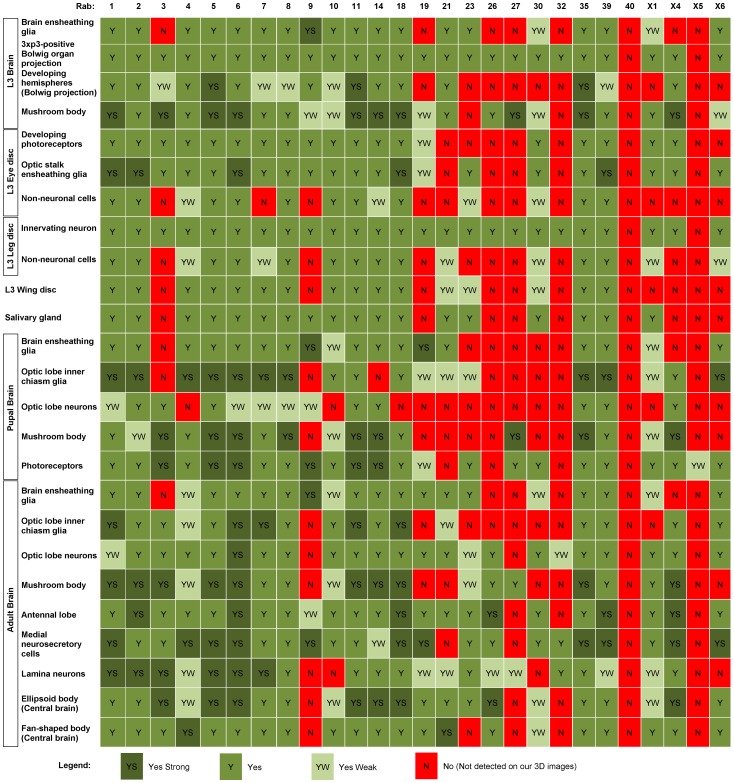
Analysis of Cellular Expression in Developing and Adult Tissues. Qualitative analysis of *rab*-Gal4>UAS-CD8-GFP expression (columns) for specific cells and tissues (rows). If expression was found to be particularly strong compared to other cells and tissues it was designated ‘YS’ for ‘Yes Strong’. If expression was found to be present but very weak it was designed ‘YW’ for ‘Yes Weak’.

With the complete profiling dataset for all *Drosophila rab* GTPases in hand, we are in a position to compare the cellular and subcellular expression data for all Rabs. In order to characterize similarities in cellular expression patterns, we identified 25 clearly discernible cell types and tissues for an assessment of the presence or absence of expression (Fig. 4). These cell types and tissues include non-neuronal developing imaginal discs, as well as neuronal and glial cell types and prominent brain structures like the mushroom bodies in the larval, pupal and adult brain. In all cases we analyzed the original high-resolution 3D confocal microscopy datasets of the *rab* genes, 23 of which were previously only qualitatively assessed [17].

To evaluate overall expression similarities, we performed pair-wise comparisons for all possible pairs of the 27 *rabs*. Absence or presence of expression was scored in a binary manner irrespective of the qualitatively different strengths of expression denoted in Fig. 4. Common presence of expression in a cell or tissue was counted towards similarity, whereas common absence was not counted. For details see Materials and Methods. The results of this binary analysis are summarized in Table 1.

The most obvious class of similarity comprises ubiquitously expressed *rabs*, including *rab1, rab2, rab5, rab6, rab8, rab11, rab35 and rab39*. A closely related second group of *rabs* comprises some potentially ubiquitous lines with larger expression variability, including *rab4, rab7, rab10, rab14* and *rab18*. Similarities between the neuron-specific or neuronally enriched lines are less obvious. This is consistent with our previous observation that the more selectively neuronally and glia-expressing lines exhibit considerable differences of their expression patterns in the brain. Indeed, only two *rabs* identified in the combined studies are expressed pan-neuronally, namely *rab3* and *rabX4*. In contrast, eight of the original 23 *rabs* are neuron- and glia-specific or strongly enriched, but they are expressed in strikingly different patterns in the brain, namely *rab9, rab19, rab21, rab23, rab26, rab27*, *rab32* and *rabX1*. Two of the four novel *rabs* added in the present study, *rabX5* and *rabX6*, fall into this category. In summary, of 27 *rab* GTPases that exhibit clear expression in the tissues analyzed here, 12 are neuron-specific or neuron-enriched; two of these are expressed pan-neuronally, and ten express in varying and surprisingly specific patterns in neurons and glial cells in the brain. The comparisons of the precise expression patterns reveal similarities that allow us to test for potential redundancies of these neuronal *rabs* not only within that group, but also with more widely expressed *rabs* that overlap in the same cell types.

### Similarities of Rab GTPases Based on Subcellular Localization Features

**Figure 5:**
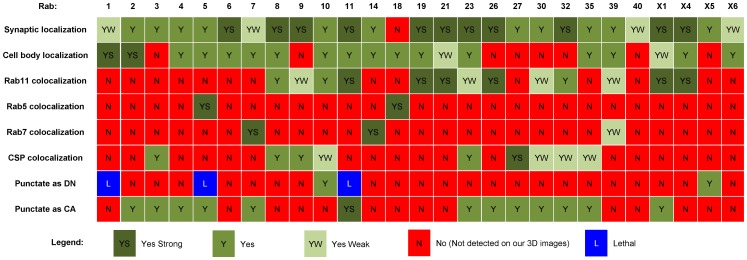
Analysis of Subcellular Localization Features. Qualitative analysis of subcellular localization features based on rab-Gal4>UAS-YFP-*rab* expression in the L3 larval ventral ganglion. ‘Synaptic localization’ and ‘Cell body localization’ indicate the presence in the respective compartments with no distinction of pre- versus post-synaptic compartments. ‘Rab11, Rab5, Rab7 and CSP colocalization’ indicate clearly recognizable individual punctae positive for both the YFP-Rab and one of the four antibody labelings. ‘Punctate ad DN or CA’ indicate whether the dominant negative (GDP-bound) or constitutively active (GTP-bound) YFP-Rab variants were clearly recognizable as distinct compartments (punctae). Labeling as in Figure 4, except ‘L – Lethal’ marks cases were L3 larvae could not be obtained because the dominant negative proteins caused lethality.

The comparison of expression patterns is not useful to identify potentially similar rab GTPases that are ubiquitously expressed. Similarly, the analysis is limited in identifying similarities amongst the differently expressed neuronal rabs. We therefore chose an independent set of more specific Rab protein and subcellular localization features for the second part of our similarity analysis. These criteria include synaptic and cell body localization, colocalization with compartments positive for Rab5, Rab7, Rab11 or CSP, and finally compartment discernibility as DN or CA variant. In all cases YFP-Rab proteins were expressed under control of their respective *rab*-Gal4 lines and analyzed in the larval ventral ganglion. A complete assessment of all 27 YFP-Rab proteins is shown in Fig. 5. Next, we performed pair-wise comparisons for binary datasets using the same rules as applied for the cellular expression data. The resulting similarities, shown in Table 2, are in many ways revealing. Several pairs exhibit 100% overlapping subcellular localization features, despite divergent expression patterns. For example, the two synaptic vesicle-associated Rab3 and Rab27 represent such a case. Indeed, both were previously shown to exhibit partial functional redundancy in secretion [29]. Moreover, several synaptic Rab11-associated Rabs, including Rab19, Rab21, and RabX4, are 100% identical for the subcellular features analyzed here. It is tempting to speculate that these Rabs may exert partially redundant functions at synapses. Several other groups await experimental verification. For example, Rab1 exhibits identical subcellular localization features to Rab6 and RabX6, even though Rab6 is mostly expressed in glial cells, as shown in this study. Similarly, RabX1 exhibits similarity to Rab1, with RabX1 restricted to neurons and Rab11 being ubiquitous.

Lastly, we compared similarities based on cellular expression patterns and subcellular localization features with primary sequence homology. In other words, we asked whether the closest *rab* homologs would also exhibit the most similar cellular and subcellular localization patterns. We highlighted the closest *rab* homologs in Tables 1 and 2. Interestingly, we found only few correlations between protein similarity and expression patterns or subcellular localization. While there are several cases where two of three criteria correlate, there is no case where all three correlate. For example, Rab3 and Rab27 are the only example that represents a pair of closest homologs that also exhibit identical subcellular localization features, but they have strikingly different expression patterns. Rab1 and Rab35 are close homologs that are both ubiquitously expressed, but these exhibit strikingly different subcellular localization features. Rab1 and Rab6 exhibit identical subcellular localization features and are both ubiquitous, but they are far apart on the phylogenetic tree of *Drosophila rab* GTPases [18]. These findings suggest that an assessment of similar functions and potential redundancies in a gene family like the *rab* GTPases may be incomplete if solely based on protein sequence homology. Our data further make numerous predictions about the potential functional properties of Rabs in multicellular eukaryotes that now await experimental verification.

## Discussion

In this paper, we present the completion and expression analysis of the rab-Gal4 kit. We identified two novel neuronal *rab* GTPases (*rabX5* and *rabX6*) and one ubiquitous *rab* (*rab30*), in line with our previous report that more than one third of *Drosophila* Rab GTPases are enriched or even specific to neurons and glia.

With the complete cellular and subcellular profiling data in hand, we could for the first time perform a systematic comparison of all *Drosophila* Rab GTPases. A key finding of this analysis shows that protein homology, expression pattern and subcellular localization in many cases exhibit revealing correlations for two of these criteria, but never for all three. In other words, we found no two Rabs that are closely related, expressed in the same pattern and mark the same subcellular compartment. This analysis may therefore provide a meaningful measure of Rab GTPase functional diversity.

Expression patterns are unlikely to correlate with protein sequence similarities, because expression is determined by regulator regions outside of the coding region. In contrast, the subcellular localization and association with compartments as a function of GTP/GDP-binding are directly related to protein functions [15], [30], yet we observed few correlations. A possible explanation for this could be that protein domains that determine the association with, for example, a distinct endosomal compartment are only short and not visible in the homology comparison over the complete protein lengths. Importantly, the cellular and subcellular localization data analyzed here provide direct experimental evidence for which *rab* GTPases potentially reside on similar compartments in the same cells at the same time – all likely requirements for potential redundancy. In contrast, the primary protein sequence is in many cases only a partial or no reliable predictor for protein structure. In this sense, the analyses presented here represent an opportunity where comprehensive subcellular localization data is available to assess the reliability of redundancies predicted by sequence homology.

The 25 cell types and tissues used for our expression analysis are not representative or comprehensive, but chosen only for discernability in the binary analysis. Hence, a similarity score of 80% based on a score of ‘20’ cannot be compared as an absolute number, but only relative to the same criteria for other *rabs*. Neither cellular expression nor the subcellular localization criteria are sufficient to assess potential redundancy. For example, both *rab3* and *rabX4* are identically pan-neuronally expressed, but Rab3 marks synaptic vesicles whereas RabX4 marks Rab11-positive compartments. Conversely, *rab21* and *rabX4* have substantially different expression patterns, yet when they overlap in the nervous system they exhibit the identical subcellular localization profile. Hence, these two rab GTPases are potential candidates for similar or redundant functions in these cells only. More generally, in the pair-wise comparison a *rab* GTPase with restricted expression (e.g. a neuron-specific *rab*) receives a low score when compared to a *rab* GTPase with broader expression (e.g. a ubiquitous *rab*), and hence will be categorized as less similar. However, this lower score does not correlate with the probability of redundancy in the cell types where the two *rab* GTPases are actually co-expressed. We therefore regard the combination of cellular and subcellular profile similarities as a means to restrict the number of potentially redundant *rab* GTPases. Importantly, all our rab-Gal4 lines represent targeting vectors for the generation of molecularly defined mutants through ends-out homologous recombination, as demonstrated in our original studies [17], [22]. Hence, the completed *rab*-Gal4 kit provides all necessary tools to experimentally test functional predictions from our analyses, as well as experiments using double and triple mutants to verify such functional relationships.
